# Most synonymous allelic variants in HIV tat are not silent

**DOI:** 10.1016/j.ygeno.2023.110603

**Published:** 2023-03-07

**Authors:** Christopher J. Giacoletto, Ronald Benjamin, Hong-Wen Deng, Jerome I. Rotter, Martin R. Schiller

**Affiliations:** a Nevada Institute of Personalized Medicine, University of Nevada, Las Vegas, 4505 S. Maryland Parkway, Las Vegas, Nevada 89154, USA; b School of Life Sciences, University of Nevada, Las Vegas, 4505 S. Maryland Parkway, Las Vegas, Nevada 89154, USA; c Center for Bioinformatics and Genomics, Department of Global Biostatistics and Data Science, School of Public Health and Tropical Medicine, Tulane University, New Orleans 70112, USA; d The Institute for Translational Genomics and Population Sciences, Department of Pediatrics, The Lundquist Institute for Biomedical Innovation at Harbor-UCLA Medical Center, Torrance, CA 90502, USA; e Heligenics Inc., 833 Las Vegas Blvd. North, Suite B, Las Vegas, NV 89101, USA

**Keywords:** Variant, Silent, Synonymous, RNA stability, Codon usage, Tat, HIV

## Abstract

The genetic code has degenerate codons that produce no change in the translated protein sequence and are generally thought to be silent. However, some synonymous variants are clearly not silent. Herein, we questioned the frequency of non-silent synonymous variants. We tested how random synonymous variants in the HIV Tat transcription factor effect transcription of an LTR-GFP reporter. Our model system has the advantage of directly measuring the function of the gene in human cells. Approximately, 67% of synonymous variants in Tat were non-silent, either having reduced activity or were full loss-of-function alleles. Eight mutant codons had higher codon usage than wild type, accompanied by reduced transcriptional activity. These were clustered on a loop in the Tat structure. We conclude that most synonymous Tat variants are not silent in human cells, and 25% are associated with changes in codon usage, likely effecting protein folding.

## 1. Introduction

The genetic code has codon degeneracies in which 1–6 codons encode each of the 20 amino acids. The different degenerate codons are synonymous, coding for the same amino acid. Since the discovery of the genetic code in 1961 by Matthaei and Nirenberg, synonymous codon that do not change a translated amino acid are generally thought to be silent allelic variants. [1] Several recent reviews cover a growing body of work that challenges this dogma, and suggests that some fraction of synonymous variants are not silent. Several reviews and genomics analyses cover the impact of silent allelic variants on disease, evolution, and address potential mechanisms of action. [2–7]

This shift in thinking is supported by the role of synonymous variants in several germline diseases. For example, synonymous allelic variants are found in the GP120 protein of many Human Immunodeficiency Virus (HIV) strains that stabilizes the RNA of the virus. [8] Approximately 8% of patients with myelodysplastic syndromes have synonymous allelic variants in *GATA2*. [9] X-linked infantile spinal muscular atrophy is a neurodegenerative disorder where patients have allelic variants in *UBE1*, including a silent variant that reaches statistical significance. [10]

Furthermore, somatic synonymous variants are beginning to appear as disease variants in cancer and this has been the subject of several recent reviews. [11–15] A survey of cancers identified >650,000 synonymous variants and with an estimated representation of 6–8% of all single nucleotide polymorphisms (SNP)s in oncogenes. [12,16] The most common is C > T transitions producing synonymous substitutions in cancer cells. [15] In liver cancer, 18% of SNPs were synonymous with common codons enriched in oncogenes, whereas synonymous substitutions for rare codons are enriched in tumor suppressors. [17] The distribution is consistent with translation efficiencies and expected expression levels.

**Fig. 1.**
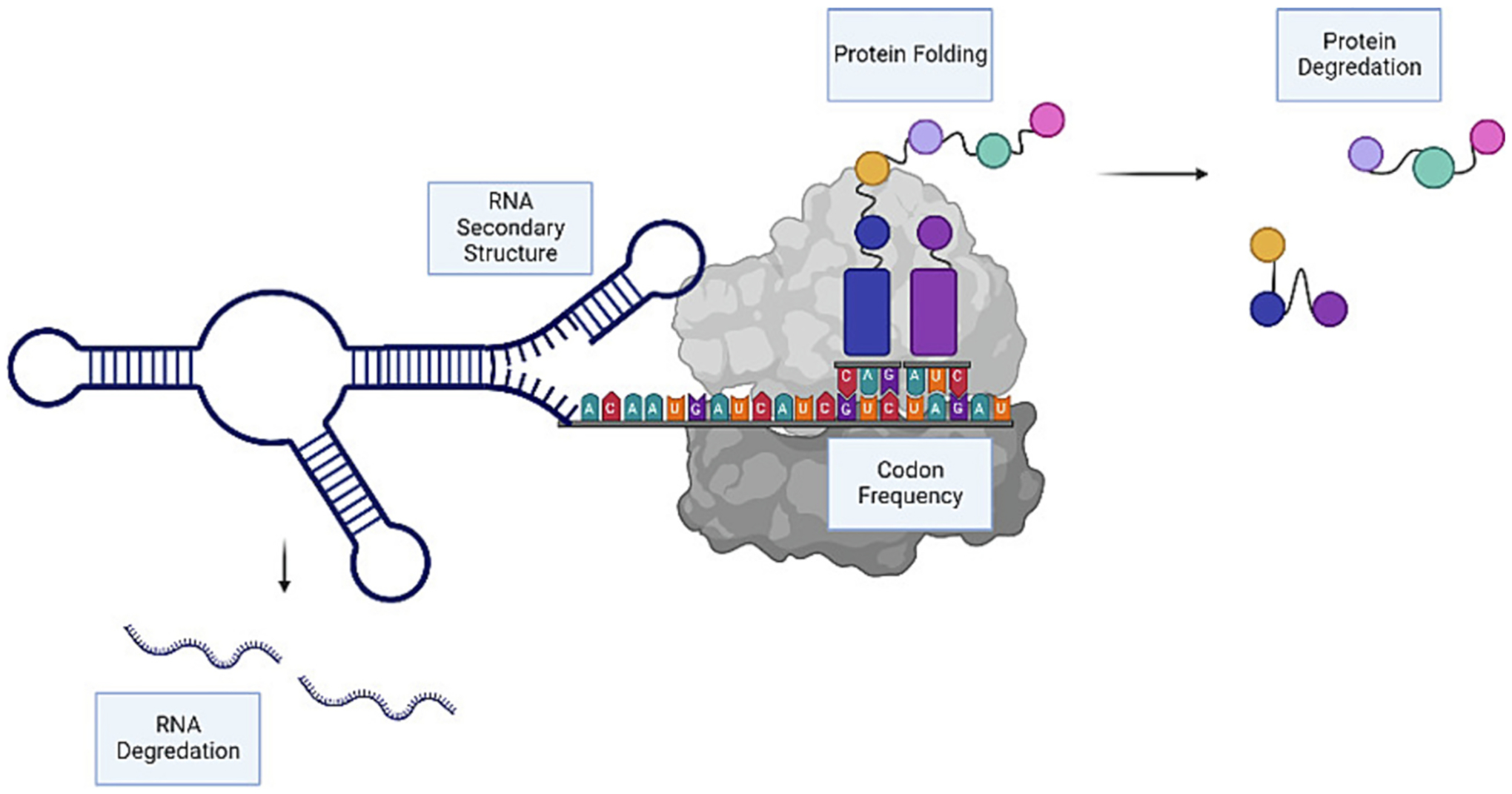
Cartoon of potential mechanisms for non-silent synonymous variants.

There are multiple mechanisms by which synonymous variants are not silent, further supporting the observed disease associations, as well as shedding new insight into disease pathology. These mechanisms include effects upon gene splicing, mRNA stability, mRNA folding and translation, protein folding, degradation and expression; and miRNA-based regulation of expression (Fig. 1). [5,14] In 1983, the concentration of rare codons in bacterial *dnaG* correlating with its relatively low expression was the first evidence suggesting that rare codons may not be silent. [18] Rare codon can slow translation, abort translational events, and produce lower gene expression. [19] Decades later, a comparison between β-actin and γ-actin suggested that more stable secondary structures in γ-actin mRNA, slowed its translation speed, resulting in co-translation protein arginylation of the nascent protein and proteosome-mediated degradation. [20] This is a potential mechanism by which synonymous variants may not be silent. The effect on RNA secondary structure on translation and protein is now further supported by many additional investigations of synonymous variants, but stronger experimental evidence supporting a role for RNA structures is needed. [2,5,21–27] Consistent with this mechanism, rare codons downstream of the signal peptide cleavage site can stall translation, improving recognition by the signal recognition particle (SRP) for translation of transmembrane proteins. [28]

Despite the growing interest in synonymous variants, it is still not known how frequently this phenomenon occurs in human cells and how often it contributes to disease pathology. In yeast, 75% of synonymous allelic variants in 21 yeast genes had reduced fitness, suggesting that this may be a frequent effect, although there is some conern with the rigor of this report. [7,29,30] Furthermore, most synonymous variant studies focus on fitness and selection, which are downstream measures of a genes function and contributing epistatic influences are more likely to confounding interpretation of mechanistic studies. Given these gaps in the understanding of synonymous variants, we investigated random sets of synonymous variants in the HIV *Tat* to determine how frequently a synonymous variant directly affects a gene’s function in cells.

## 2. Results

We recently developed a new assay system called the GigaAssay that measures the functional activity and effect of 1000 s of mutants in a gene. The GigaAssay was used for two experiments on Tat examining the transcriptional activities of all possible single amino acid substitutions in two different cell lines. [31,32] For each mutant, the activities were measured in ~ 100 separate single cells and the measured activities had a ~ 95% accuracy measured by three independent approaches. All experimental methods and the bioinformatics pipeline details are in these previous reports. Because each cDNA was independently barcoded with a unique molecular identifier (UMI), we were able to separately analyze cDNAs with altered allelic variants when compared to reference arising from errors in oligonucleotide synthesis of the saturating mutagenesis libraries. These errors arise because coupling efficiency’s during oligonucleotide synthesis are typically 99.6%–99.8% and thus, many synthesized oligonucleotide molecules contain errors. These errors can be considered generally random, but are prone to limited biases arising from the oligonucleotide coupling reactions. Our experiment had 179,673 unique UMIs with >2.5 reads per million (RPM). In the original, report, this dataset was filtered to discard reads that had synthesis errors, including any synonymous variants. Synonymous allelic variants were extracted and analyzed to estimate their frequencies being either silent or not.

There are three important considerations for this application of the dataset: 1, the GigaAssay is not a screen, rather it is an assay where both positives and negatives are directly measured with high accuracy and negatives are not inferred [33]; and 2, since the synonymous variants arose due to synthesis errors, there were fewer barcodes with UMIs than the >100 UMIs for each allelic variant previously reported, typically <6 for each mutant. Therefore, we only analyzed mutants that had at least 2 barcodes in at least one of the replicates and activities were averaged for technical replicates (File S1). We also analyzed the unfiltered dataset and the results generally agreed (File S2). Thus, the unfiltered data are suitable for estimating the rates of synonymous mutants that are silent or not.

### 2.1. Synonymous variants on a WT tat background

**Fig. 2.**
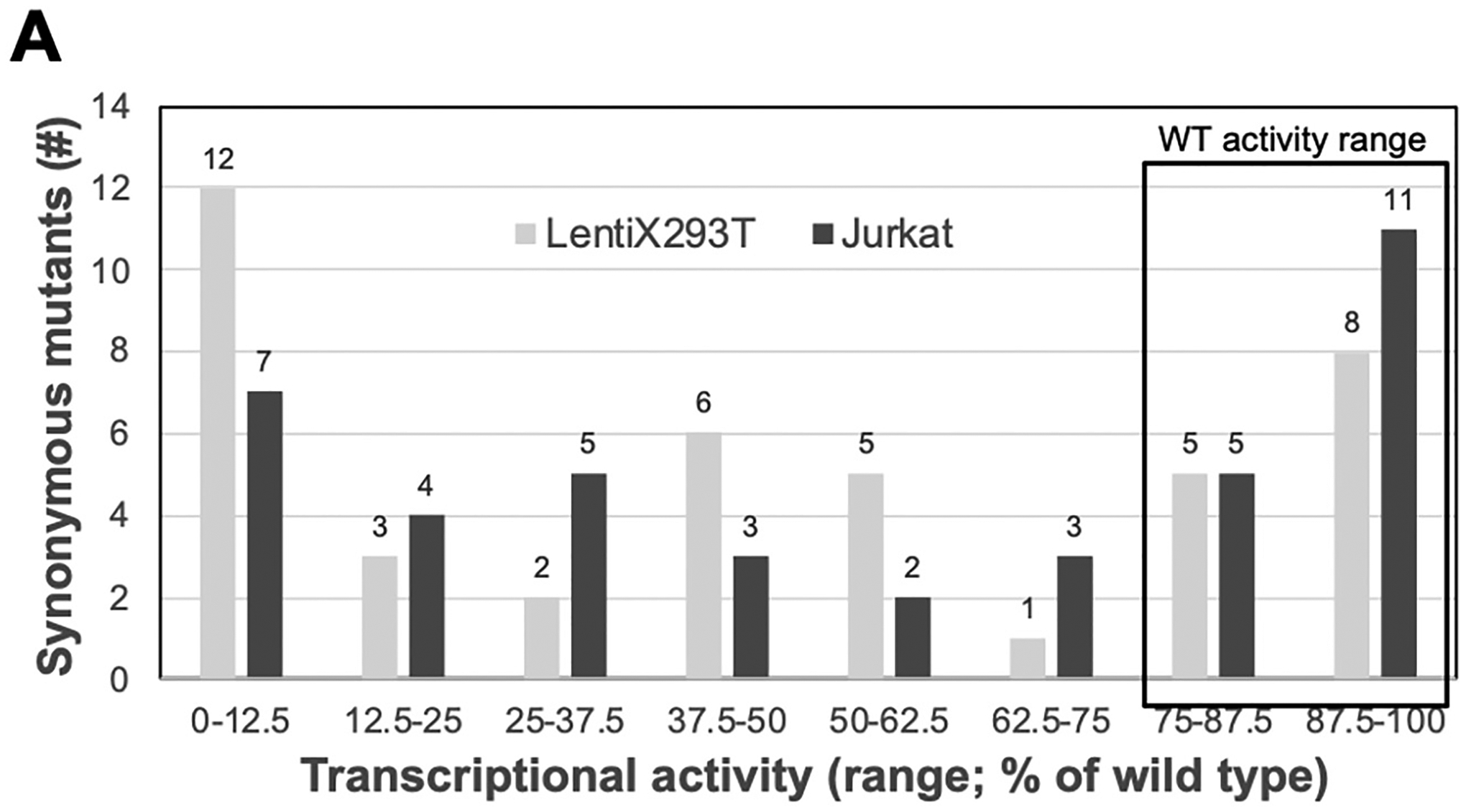
Transcriptional activity of WT Tat with synonymous variants. Histogram of the transcriptional activities for synonymous variants in LentiX293T and Jurkat cells. The box indicates mutants classified as WT activity.

There were 42 synonymous variants analyzed in LentiX293T cells, with 31% being silent with wild type (WT) activity (Fig. 2, File S1). cDNAs with UMIs and activity score above 75%, were classified as WT, and those below 75% are reduced activity. We used this threshold because the average activities levels of UMI-coded WT cDNAs were 75% of maximal WT activity (*n* = 14,886 for 2 replicates in each of LentiX293T and Jurkat cell lines). When these mutants were not filtered (*n* = 86), similarly, 41% were silent with WT activity (Fig. S1, File S2). Similar measurements of 48% of synonymous variants with WT activity were observed when the same experiment was performed in Jurkat cells (*n* = 40), respectively (Fig. 2). Approximately 25% of the synonymous mutants produced the similar activities in both LentiX293T and Jurkat cells. The identification of the same random variants in both cells was expected because they were from the same variant library.

**Fig. 3.**
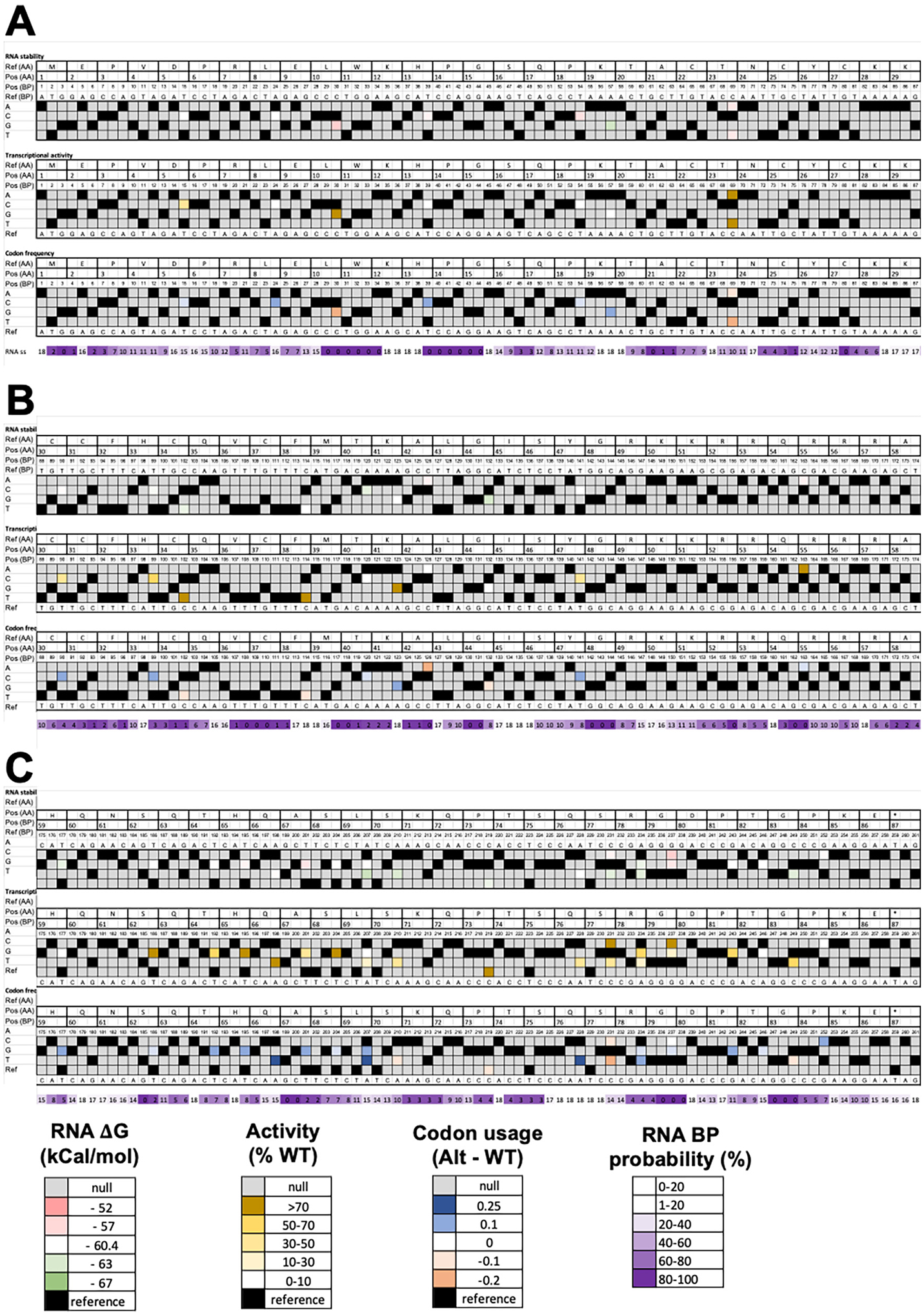
Heatmaps for synonymous variants in LentiX293T cells. Heatmaps showing different metrics for all nucleotides and amino acids positions: (A) amino acids 1–29; (B) amino acids 30–59; and (C) amino acids 59–86 and stop codon for Tat. The Tat-driven transcriptional activity (middle panels); ΔG for the Tat mRNA secondary structures (top panels); and the difference between the human codon frequencies for mutant and WT Tat (bottom panels) are shown. The double strand RNA probability score calculated from the percent of 18 lowest energy structures from Mfold having base pairing is shown for each nucleotide position (purple gradient; below bottom panel). A color key is shown for each heatmap. Heatmaps for synonymous variants in Jurkat cells are in Fig. S2. A key is provided for each row in the heatmap.

To better understand the distribution of silent variants and if there were any obvious patterns, we plotted transcriptional activity on a heatmap (Fig. 3, white to brown heatmap). The heatmap shows minor local clustering where groups of mutants with either WT activity or loss-of-function (LOF) activity are in the same region of the RNA. In these experiments, WT refers to the Tat sequence from pNL4–3 genome sequence (GenBank: AF324493.2). Similar results were observed in Jurkat cells (Fig. S2).

**Fig. 4.**
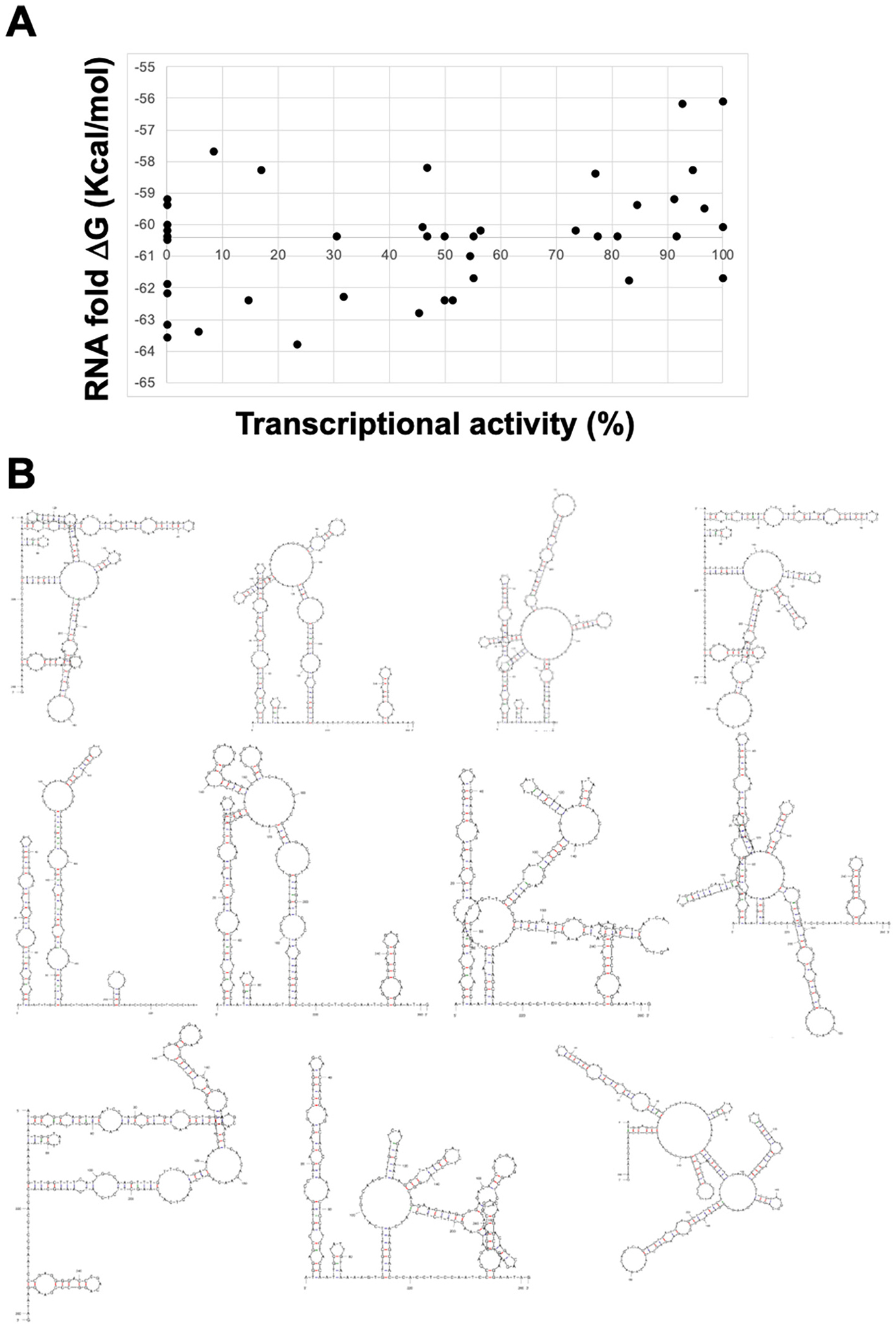
Effect of predicted mRNA stability on transcription activity in LentiX293T cells. A. Transcriptional activity vs. mRNA secondary structure ΔG scatter plot for synonymous Tat mutants. The lowest ΔG for the WT Tat RNA calculated with Mfold is −60.4 Kcal/mol. The data fits a trend line with R^2^ = 0.14. A similar scatter plot for Jurkat cells is in Fig. S3a. B. RNA secondary structures for the 11 lowest Tat mutant RNA ΔGs is shown.

We investigated potential mechanisms by which synonymous variants were not silent. Since there was potential clustering of silent variants with either WT or impaired activity along the DNA sequence, we examined how synonymous variants effect the predicted RNA secondary structure and stability based on Watson-Crick base pairing. The predicted ΔG of RNA structures for WT and each *Tat* mutant were calculated with Mfold on the QuikFold server [34]. A ΔG was calculated for each synonymous mutant RNA. In LentiX293T cells, there was only a weak correlation (R^2^ = 0.14) between the ΔG for the synonymous mutant stability and levels of transcriptional activity. The positive correlation suggests that synonymous mutants with predicted less stable RNA had a weak trend to have more activity (Fig. 4a). This trend was also not strong when mutants in Jurkat cells were analyzed (Fig. S3a), nor when plotted on a heatmap [Fig. 3 (green to red heatmap)].

Since RNA secondary structure is one of the reported mechanisms for synonymous variants not being silent, we further assessed any potential contribution of RNA secondary structure to the *Tat* transcriptional activity. We calculated the RNA base pairing probability from an ensemble of the 11 lowest energy predicted structures for WT Tat RNA using Mfold (ΔG range = −60.4 to −57.4 kcal/mol). Comparison of the base pairing probabilities to the transcriptional activity (Fig. 3, white to purple heatmap) again reflects no obvious pattern where variants in high probability stems or high probability loops correlate with a consistent change in transcriptional activity. Collectively, while there may be variants with single amino acid substitutions that impact activity due to RNA secondary structure, this does not appear to be a major mechanism by which synonymous variants are not silent.

**Fig. 5.**
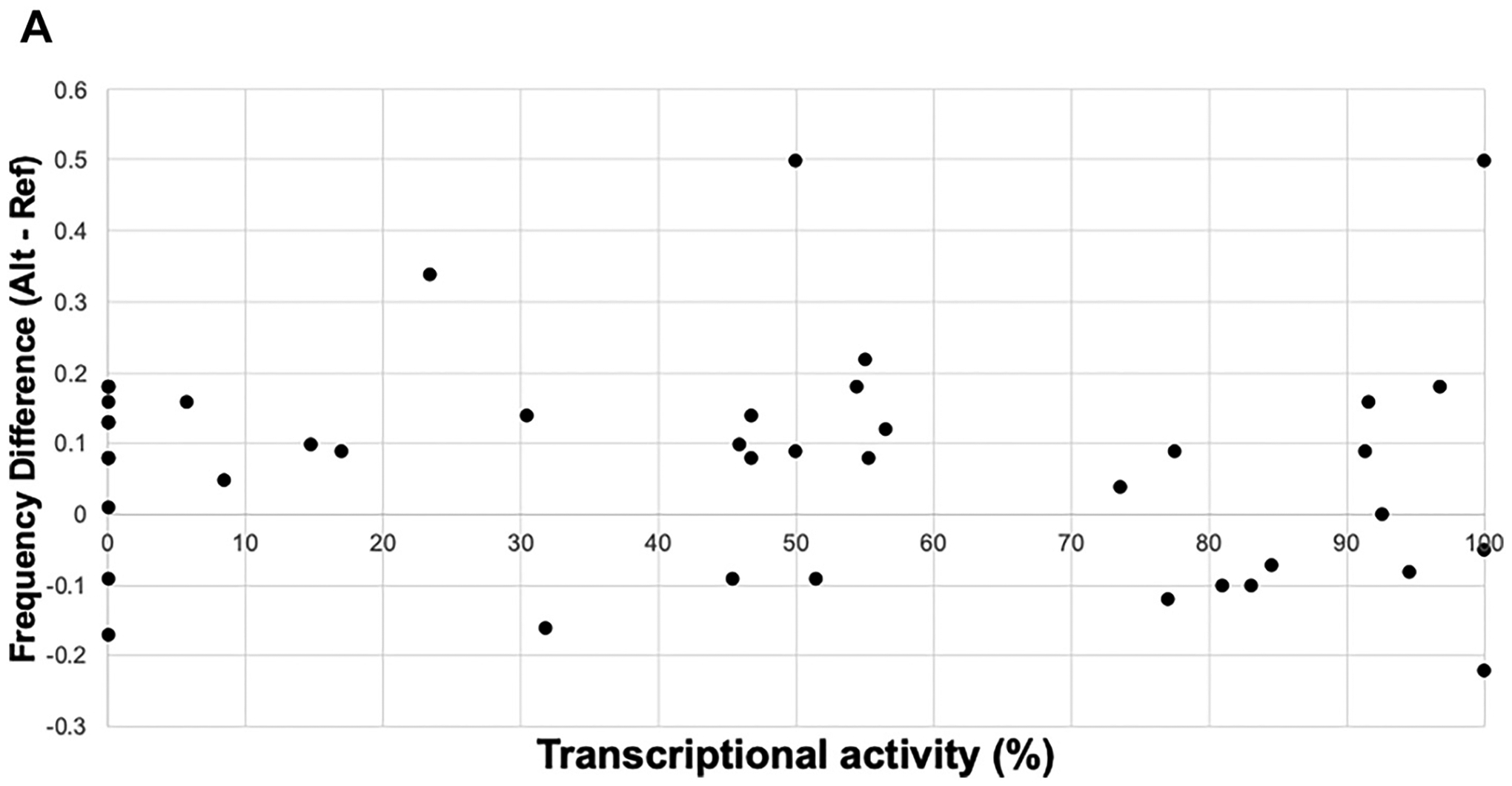
Effect of codon usage on transcription activity in LentiX293T cells. A scatter plot of transcriptional activity vs. the difference in codon usage when compared to WT Tat RNA. A similar scatter plot for Jurkat cells is in Fig. S3b.

Another potential mechanism that produces non-silent synonymous variants is mutation to rarer codons that can affect either gene expression or protein folding, either of which can impact Tat-driven transcriptional activity. We determined if the change in codon usage frequency for synonymous variants correlated with changes in transcriptional activities. We used the human codon frequency table because the experiment was in human cells lines. Linear fitting to the data showed no trend or correlation in LentiX293T (R^2^ = 0.01; Fig. 5) or Jurkat cells (Fig. S3b). However, the heatmap organizing activity to codon usage frequency across the protein sequence showed some clusters and regions of correlation [Fig. 3 (blue to orange heatmap)]. Between residues 8–23 there were 3 LOF synonymous variants that also had large increase in codon usage, whereas 2 synonymous variants with WT activity had a decrease in codon usage frequency. The same trend was observed over residues 30–40 all though the reduced activity was less pronounced.

**Fig. 6.**
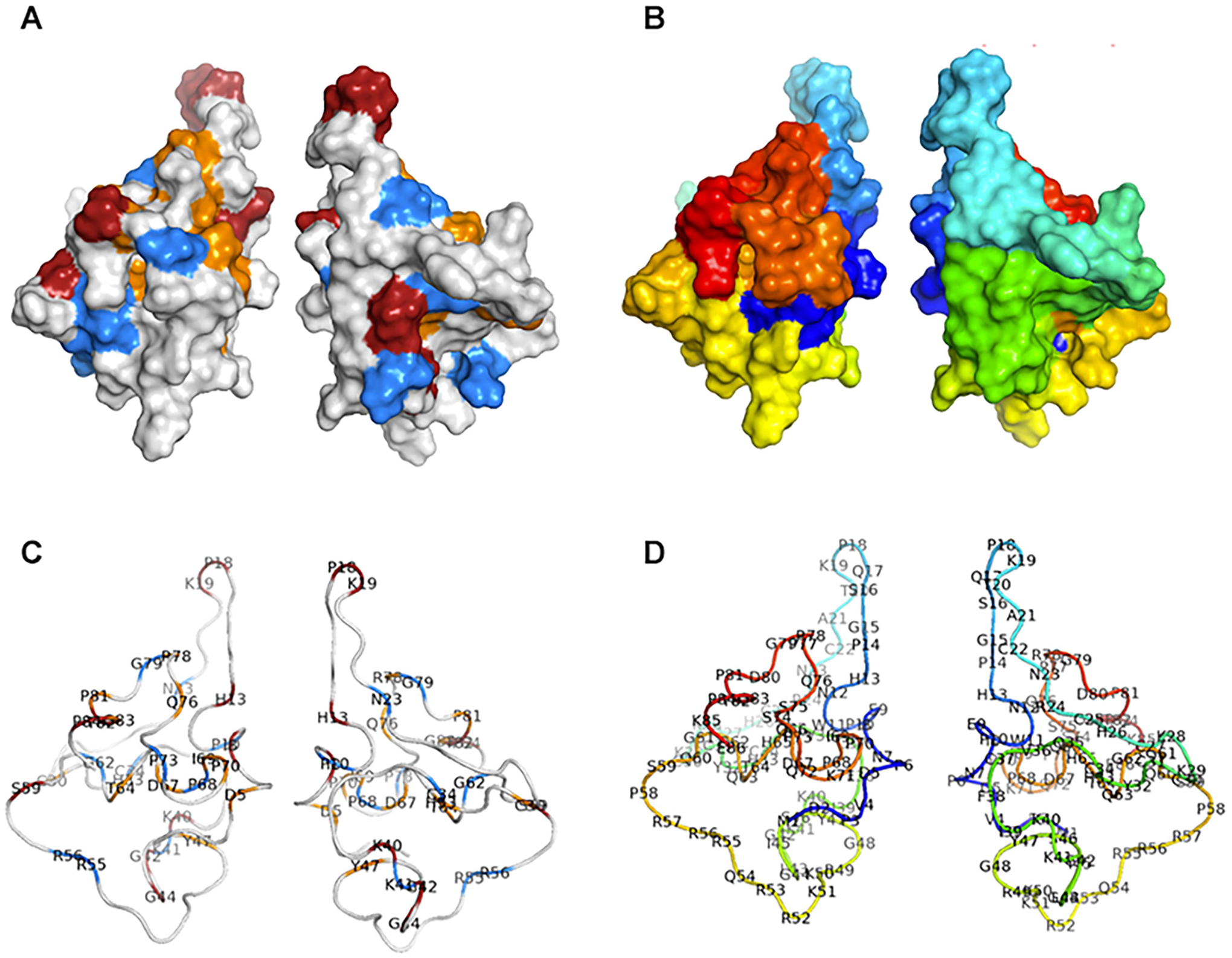
Three-dimensional location of synonymous variants and impact on activity on a Tat structure surface plot. A. 3D structure surface plot of Tat with residues colored by the transcriptional activity of synonymous variants. Blue = WT activity; orange = reduced activity; and red = LOF activity B. 3D structure surface plot of Tat with residues colored in a rainbow by order of positions using PyMol. C. Ribbon plot of Tat with residues colored by the transcriptional activity as in A. Residues with measured activity are labeled by position and single letter code. D. Ribbon plot of Tat with residues colored by order of amino acids as in B. Residues are labeled by position and single letter code.

The localized effect of changes in codon usage suggests that the effect on at least some synonymous variants is at the level of translation or protein synthesis, rather than effecting the RNA. Therefore, we plotted the transcriptional activity of synonymous variant positions onto a surface map of the 3D structure of Tat (PDB: 1TIV). [35] This revealed a 3D spatial cluster of residues (positions 13, 18, 19, 76, 78, 81) that have LOF or reduced activity (Fig. 6). These residues all had variants with increased codon usage frequencies (Fig. 3). These and other clusters were not as obvious on a heatmap and there was no correlation when compared to our previously reported saturating missense mutagenesis heatmap which also maps other protein features such as function, post-translational modifications secondary structures, and surface accessibility (Fig. S4). [31] Collectively, this analysis supports a mechanism for about 25% of synonymous variants producing more used codons, likely effecting translation velocity and changes to folding or expression.

## 3. Discussion and conclusions

There is growing interest and publications exploring the effects of synonymous allelic variants. We view three aspects of our work being unique. The first is that we have examined a random sampling of synonymous variants to determine how they affect Tat-driven transcription of a fluorescent reporter. This is the first measure of a randomly sampled set of synonymous variants in a gene, measuring their impact on a molecular function in human cells. Thus far, all studies examining potential changes for synonymous variants have measured either changes in fitness or translational velocity that effects gene expression and most are on microbe systems.

The second is our random sampling of silent variants in Tat estimating that ~59–67% of synonymous variants are not silent with respect to transcriptional function. We were surprised at this result, but note that it is consistent with a recent analysis of yeast in which 75% of 1866 synonymous variants in yeast were not silent, having altered fitness. [31] These findings has rather extreme implications in several areas of biomedical science, which are discussed below.

The third is that most of the variants do not appear to use one of the previously reported mechanisms. Only 25% of variants appear to have reduced codon frequencies that likely effect expression or folding as supported by clustering on a loop in the 3D structure of Tat. The remaining 75% did not correlate with metrics used to assess other published mechanisms.

There are now a growing number of examples where synonymous variants are associated with a disease. The dbDSM database has 2548 instances of deleterious synonymous variants of which 1295 are associated with a disease. However, 536 of these annotations are from the genome wide association study (GWAS) catalog or database and cannot be readily distinguished from passenger variants arising from linkage disequilibrium. [36] Nevertheless, clearly synonymous variants are a new potential disease mechanism that may be more pervasive than previously considered. Our report of a high rate of synonymous variants that are not silent among a randomly sampled subset, suggests that geneticists should consider synonymous variants as a potential mechanism in their investigations of disease pathology and diagnostics. Our finding expands upon a recent report that 75% of synonymous allelic variants in 21 yeast genes are not silent. [7]

There are several other experiments that support a functional role for synonymous variants. For example, several synonymous variants increase organism fitness. During in vivo evolution of Methylobacterium, bacteria gained synonymous variants producing slightly increased fitness. [37] *AraA* gene synonymous variants effect bacteria fitness. [38] Silent variants in the HIV genome effect viral fitness. [39]

### 3.1. Mechanisms

While there are a growing number of examples where synonymous variants affect diseases, evolution, fitness, and epistasis, there is no clear mechanism that can be ascribed to all synonymous variants. [3] Our model system investigating the transcriptional reporter activity from Tat expression offers an opportunity to more directly measure effects on Tat’s mechanistic role to increase transcription, ruling out epistatic influences that could arise during a fitness selection screen.

From a general perspective, synonymous variants could influence synthesis or degradation of the Tat RNA or protein. This would include steps of Tat transcription, pre-mRNA processing, export of the mRNA to the cytosol, folding and potential degradation of the mRNA, recruitment of the RNA to the ribosome, translation, protein folding and co-translational degradation. Synonymous variants could potentially influence miRNA binding or other functional properties of the mRNA. If properly folded, once the protein is released from the ribosome, it should not differ from WT protein.

An advantage of our experiments is that we can compare all ~40 random synonymous variants to each other to assess how often one mechanism might be used when compared to other potential mechanisms. The are several reports where synonymous variants act before or at mRNA recruitment to the ribosome so, we started by examining the RNA stability. However, while we could identify a few examples where a more stable RNA was predicted and produced reduced activity (positions 59, 70, 77, 78, and 83) the effects were generally modest, mildly reducing Tat activity. Notably, there was an LOF allele at position 19 that had a 3 kCal/mol reduction in RNA stability. The variants we examined were all single base substitutions and are only a minor portion of the much larger mRNA. A similarly modest effect was observed when RNA base pairing probability was examined.

The results suggest that the effects of alternative codons on calculated RNA stability or secondary structures are not the sole major mechanism by which some synonymous variants are not silent. However, we must consider the caveat that there is in an ensemble of predicted Tat mRNA structures with similarly low energy. [40] Single variants could influence which and how frequently the low energy structure are sampled. Different structures could be more prone to binding a protein or degradation. It is possible that this is a limitation of Mfold, which focuses on Watson-Crick base pairing and are predictions. There are much stronger levels of evidence for RNA structure that could be explored, thus effects at the RNA level would require a much more in depth study of the synonymous variants that we tested. [40] The metrics examined here are not experimental and may not probe more specific regional effect well, although this is somewhat addressed by the general lack of any strong pattern in the heatmaps we produced. Perhaps analysis of a larger experimental dataset could also reveal patterns that were not evident.

Co-translational effects on protein elongation are other mechanisms by which synonymous variants are not silent. We did observe 25% of the synonymous variants clustered in a loop near the N-terminal end of Tat had reduced or LOF alleles, associated with a large increase in codon usage over the WT sequence. We do not know why we saw a deleterious impact with increased codon usage in the synonymous mutant, whereas most other reports have LOF caused by rarer codons. For example, suboptimal codons in the N-terminus have larger effect on expression through reduced secondary structures and codons are also a determinate for mRNA stability. [5,6] One explanation could be adjacent codon interactions during translation which has been observed and reviewed by Brule [5] Nevertheless, codon usage in WT genes seem to be important as rare codons are conserved across species. [26,41]

Since several of the synonymous codons producing LOF activity localized to a loop in the 3D structure, it seems likely that these synonymous mutants alter protein folding. We presume that with more frequent codons increase translation velocity too much, and proper folding of the nascent chain is not consistent with the increased velocity. This would likely produce incorrectly folded protein and degradation, but these ideas will need to be further assessed. Recently a codon usage code for co-translational protein folding was suggested that could include negative influence from increases in translation velocity. [4] Another consideration that silent variants may elicit stronger effects in double mutant haplotypes that produced intragenic epistasis and effect translation velocity as seen in *CTFR* mRNA for cystic fibrosis. [13]

Another proposed mechanism is altered alternative splicing due to synonymous variants. In this example, KRas c.Q61K is an pathogenic allelic variant by itself, but alleles with an additional c.G60G variant produces a cryptic splicing site. [42] We examined the Tat sequence for different synonymous variants that effected activity, but we did not observe any pattern, suggesting that splicing only plays a minor role, if it does occur.

Now that our study has defined a set of synonymous variants in Tat that are not silent, this lays the foundation to further explore mechanism of actions. Our survey of potential mechanisms suggest that the effect is at least partially after the protein is being translated. However, there is no potential known mechanism for at least half of the synonymous variants. Some obvious next steps will be to explore how these variants effects translation velocity, protein folding, co-translational degradation, protein expression levels, or some of the other possible mechanisms that have yet to be explored such as RNA export.

### 3.2. Limitations and perspective

There are several limitations in our experimental design. We do not think that any impact our conclusions, but readers should be aware of these nuances. First, most studies that investigate the impact of synonymous variants examine effects upon fitness. In this study, we examined how a viral gene induces transcription of a fluorescent protein reporter in a human cell line. Our model has the advantage of examining a direct function of the gene, rather than the downstream measurement of organism or cell fitness. However, our model is limited because we are studying a viral gene and use a reporter cell line, in which the results could possibly not translation to other systems. However, since the 75% rate of synonymous non-silent variants was similar to a recent study on genes that effect yeast fitness, we suspect this is unlikely to be a problem. [7]

We suggest that scientists carefully consider deviations from the use of a reference sequence for experimentation, even in in the context of optimizing expression of proteins as some synonymous mutations can significantly alter properties of these proteins (e.g. folding), which in many cases are produced with the intention of being used for therapeutics. Synonymous mutations introduced into a codon optimized gene may have caused the elimination of particular mechanisms, such as miRNA binding sites or changes to folding structure that were originally introduced through codon optimization.

Our experiment assumes that the synonymous variants in the Tat sequence were randomly sampled. While oligonucleotide synthesis is not random, most synthesis errors are deletions, that would unlikely impact our results since they would be filtered and discarded. [43] As with a pipeline that uses next generation sequencing (NGS), there are many decisions with filtering, that could impact the results. However, there is no clear patterns in the synonymous variants produced in our experiment supporting little, if any bias, and a random sampling.

Since we observe errors in our UMI barcodes, we use a barcode grouping step that relies on matching UMIs with a Levenshtein of 2. [44] We chose this distance considering the length of our barcodes and the probability that we should not observe and incorrect groupings. Our dataset had 17 separate samples including, libraries from different steps of the GigaAssay with technical and biological replicates. We required that after demultiplexing, each UMI barcode be observed in at least two of the libraries and have the same variant call to rule out any sequencing errors, as it is unlikely that the same error would independently occur in the same UMI twice. We tested simulations with different thresholds for the number of UMI barcodes per mutant and the results did not significantly change, thus the observed errors do not affect our conclusions.

## 4. Methods

*Measuring the Tat-driven transcriptional activity of synonymous variants with the GigaAssay*. All experimental methods were as previously described using the Tat cDNA (accession number: AAK08486.1). [31,32]

### 4.1. Bioinformatics

Data analysis was completed using the raw data from the HIV-1 Tat GigaAssay. Transcriptional activities were calculated as previously reported [31,32]; however, we used additional measures of UMI barcode filtration. The read data were from 17 samples including the plasmid library, selected cell libraries, and flow-sorted pools for each of two cell lines. UMI barcodes were retained only if they had the same variant call in 2 or more of the 17 samples.

The experimental data were from 2 biological replicates using LentiX293T and Jurkat cells, each with 2 technical replicates. Mutants were filtered such that at least one of the 4 replicate analyses were required to have 2 or more UMI barcodes. This filter estimates an approximate 30% error rate in LentiX293T cell transcriptional activity scores and a 34% error rate in Jurkat cells. An error is defined as replicate measures for mutant having an activity difference >30%. This was calculated by taking the known activities of Tat from the GigaAssay, and randomly sampling barcodes to the amount that matched the distribution of the data in the synonymous mutants. If the resulting activity for each mutant was different from the activity calculated by using all the barcodes by >30% it was counted as an error.

Transcriptional activities were calculated from the RPM of the high-green fluorescent protein (GFP) pool divided by the sum of the RPMs from the high-GFP and low-GFP pools. Activities were then averaged for technical replicates for all figures and compared across cell lines. Codon frequencies were investigated as a potential mechanism for LOF in synonymous variants. Human codon frequencies were taken from an online resource GenScript. [45] Each mutant’s difference in codon frequency (alternative (Alt) vs. reference (Ref)) was calculated.

### 4.2. RNA structure analysis

Predicted RNA stability, RNA secondary structures, and base pairing probabilities were calculated with Mfold and QuikFold. [34] To calculate single strand RNA propensity, the RNA secondary structures with the lowest 18 ΔGs were calculated with Mfold (−64 kcal/mol to −62 kcal/mol). The single strand probability is the percentage of the 18 structures with the nucleotide being single stranded. The ΔGs resulting from the introduction of a mutant sequence were calculated using default parameters of QuikFold RNA v4.

### 4.3. Figure preparation

Histograms, scatter plots, and heatmaps were prepared with Excel. Cartoons were created with BioRender. RNA secondary structures were created with Mfold. [34] 3D surface plots were made with PyMol. [46]

## Supplementary Material

1

2

3

## Data Availability

All data is shared as part of a previous submission or is included.

## Abbreviations:

Alt: alternative sequence
GFP: green fluorescent protein
GWAS: genome wide association
HIV: human immunodeficiency virus
LOF: loss-of-function
LTR: long terminal repeat
Ref: reference sequence
RPM: reads per million
SNP: single nucleotide polymorphism
SRP: signal recognition particle
UMI: unique molecular identifies
WT: wild type
