# Supplementary material for: Most synonymous allelic variants in HIV tat are not silent
Source: Genomics. Author manuscript; Available in PMC 2023 Jun 11. (PMC10257815; doi:10.1016/j.ygeno.2023.110603)
Supplement: 1 [file NIHMS1903390-supplement-1.pdf]

A

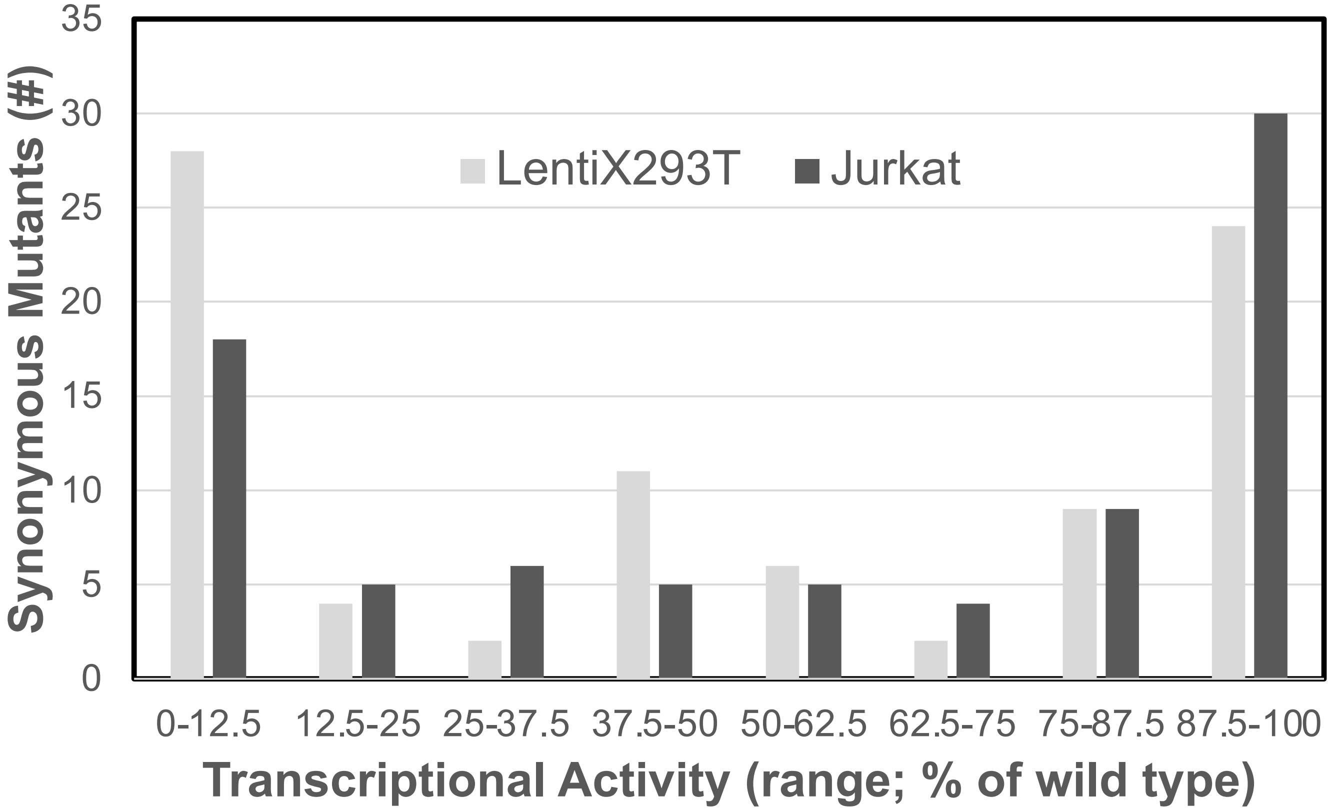

Figure S1. Histogram for synonymous mutant activity ranges in LentiX293T and Jurkat cells for unfiltered data. Histograms of the transcriptional activities for synonymous mutations in LentiX293T and Jurkat cells for unfiltered data.

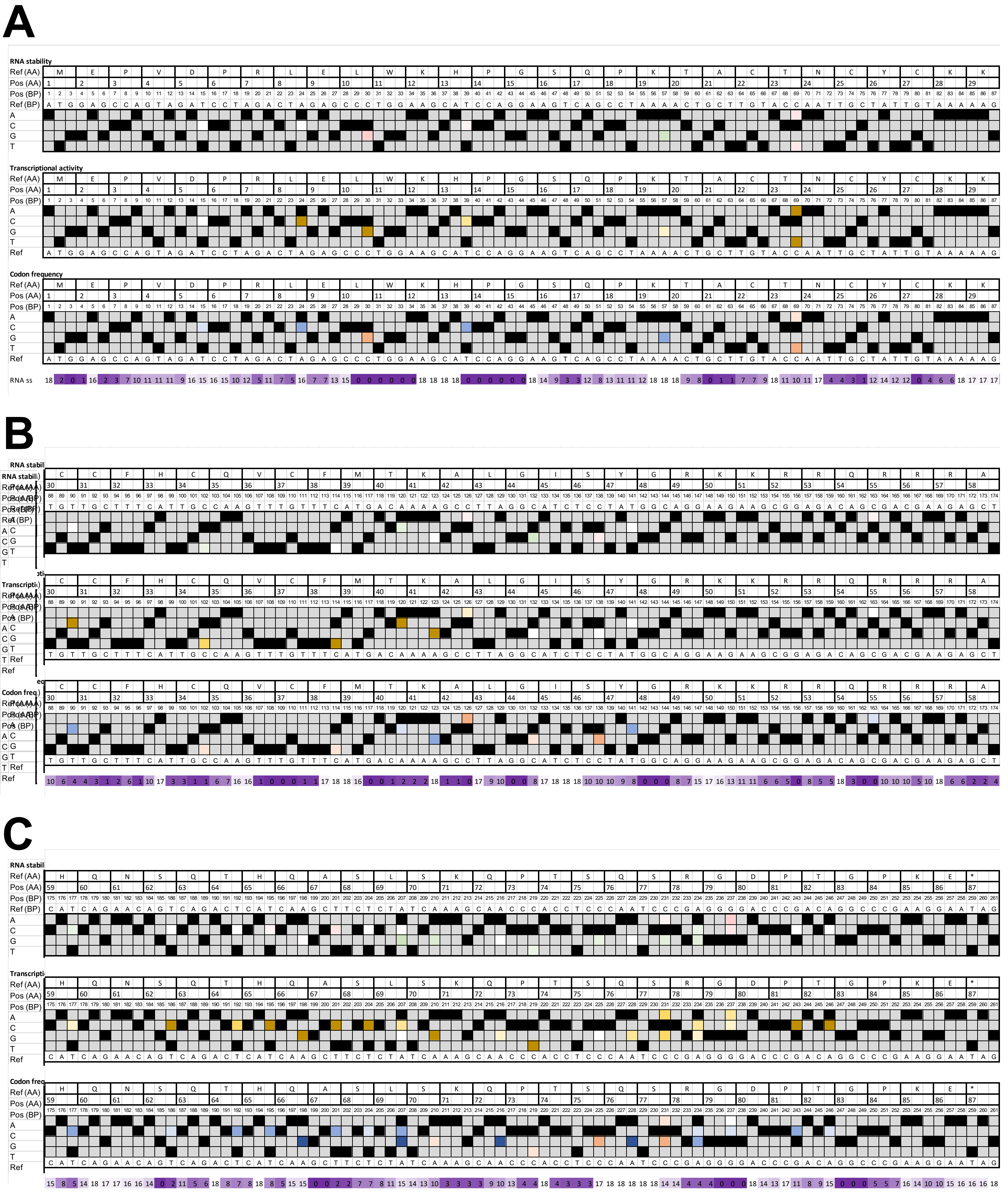

Figure S2. Heatmaps for synonymous variants in Jurkat cells. The Tat-driven transcriptional activity (middle panels); ΔG for the Tat mRNA secondary structures (top panels); and the difference between the human codon frequencies for mutant and WT Tat (bottom panels) are shown. The double strand RNA probability score calculated from the percent of 18 lowest energy structures from Mfold having base pairing is shown for each nucleotide position (purple gradient; below bottom panel). The organization and color key are the same as in **Fig. 2**.

A

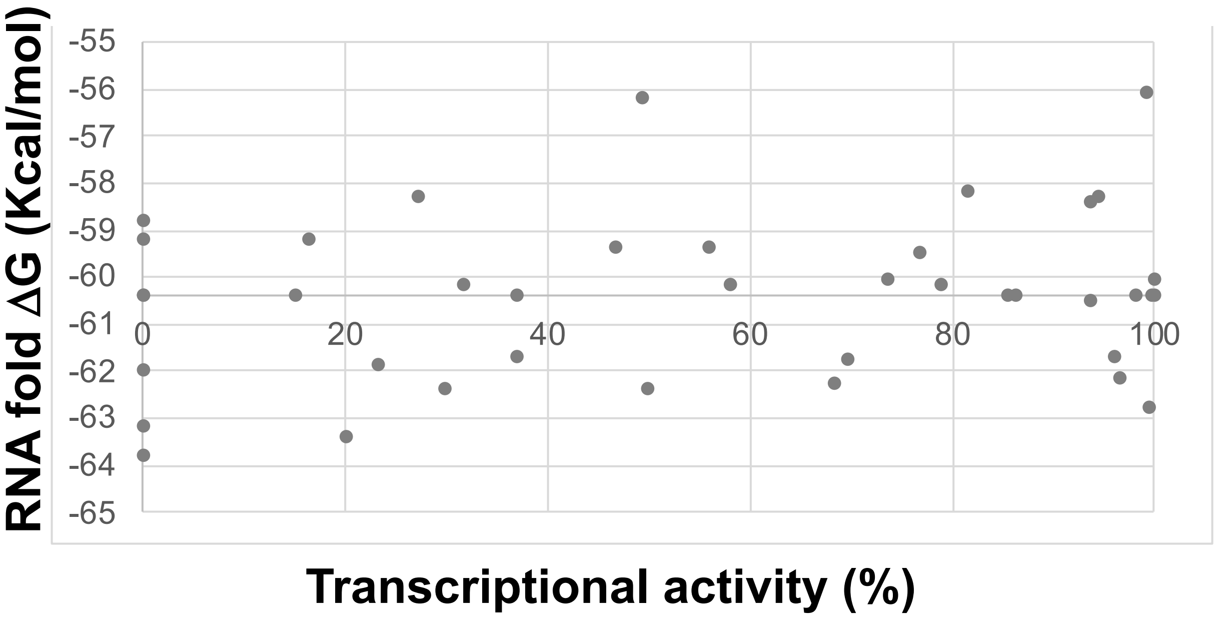

B

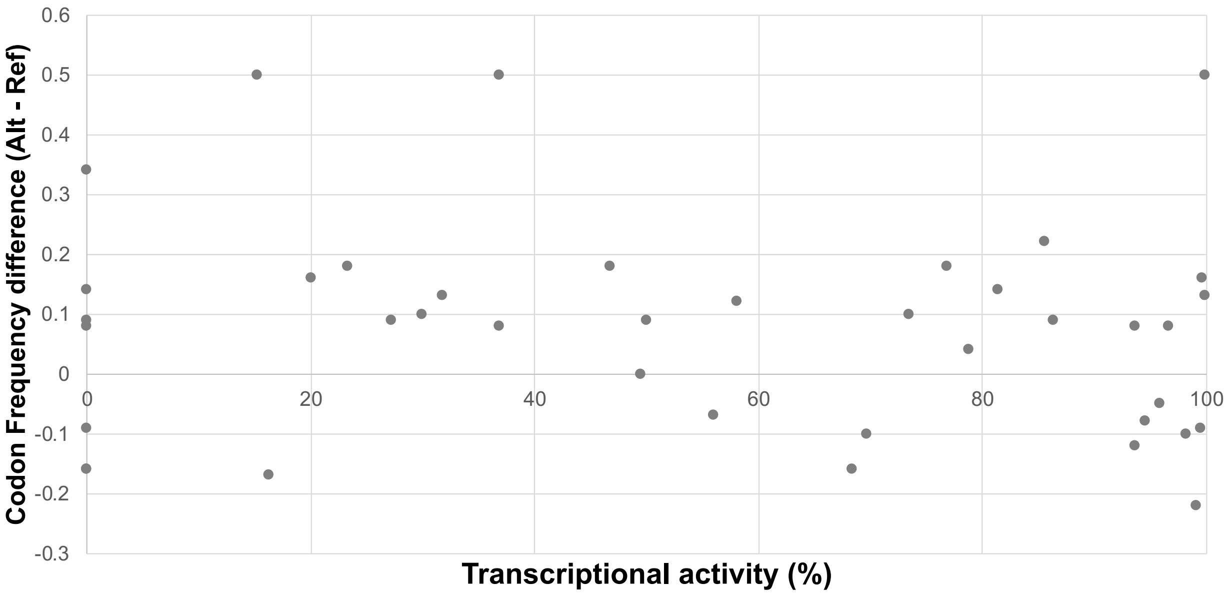

Figure S3. RNA stability and codon usage effect on activity in Jurkat cells.
A. Effect of predicted mRNA stability on transcription activity in Jurkat cells. **A.** Transcriptional activity vs. mRNA secondary structure ΔG scatter plot for synonymous Tat mutants. The lowest ΔG for the WT Tat RNA calculated with Mfold is -60.4 Kcal/mol. **B.** Effect of codon usage on transcription activity in Jurkat cells. A scatter plot of transcriptional activity vs. the difference in codon usage when compared to WT Tat RNA.

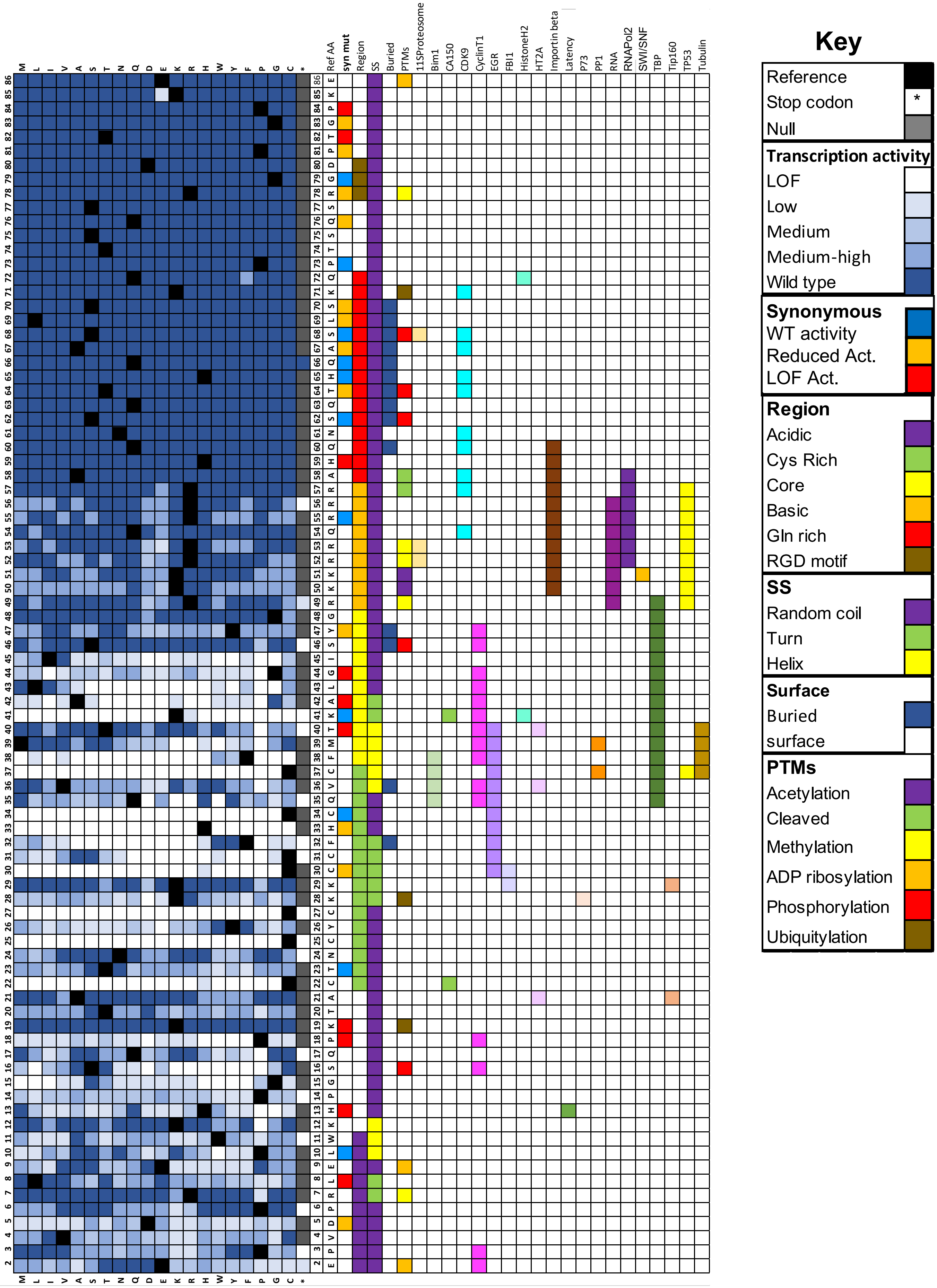

Figure S4. Heatmap comparing Tat-driven transcriptional activity of missense mutation in LentiX293T cells as previously reported with the activity of silent mutations added. A color key is shown.
